# Understanding medical aspects of violent crimes in Sweden’s region Skåne: a retrospective cross-sectional design of the ViCS project

**DOI:** 10.3389/fpsyt.2023.1287007

**Published:** 2023-11-09

**Authors:** Alexandra Ringqvist, Basem Aloumar, Carl Johan Wingren, Ulf Ekelund, Ardavan M. Khoshnood

**Affiliations:** ^1^Department of Clinical Sciences Lund, Emergency Medicine, Skane University Hospital, Lund University, Lund, Sweden; ^2^Department of Clinical Sciences Lund, Lund University, Lund, Sweden; ^3^Unit for Forensic Medicine, Department of Clinical Sciences Malmö, Faculty of Medicine, Lund University, Malmö, Sweden; ^4^Department of Forensic Medicine, Copenhagen University, Copenhagen, Denmark; ^5^Department of Clinical Sciences Malmö, Emergency Medicine, Lund University, Skane University Hospital, Malmö, Sweden

**Keywords:** homicide, criminals, gun violence, retrospective studies, Sweden, cross-sectional studies, violence, hospitalization

## Abstract

**Introduction:**

While there has been a reduction in specific homicide categories in Sweden, the last decade has witnessed an increase in the overall rate. The escalation is predominantly linked to heightened gun violence associated with criminal gangs. As a result, Sweden faces an extreme rate of shootings and firearm-related homicides, constituting one of the most severe instances across Europe. However, comprehensive scientific studies on this phenomenon are lacking. This paper presents the design of the Violent Crimes in Skåne (ViCS) project, which aims to investigate violent crimes requiring hospitalization or causing death in Sweden’s region Skåne from a medical, forensic, and criminological perspective. The project aims to examine the epidemiology and trends of violent crimes, injury profiles, treatments, patient outcomes, causes of death, and victim demographics.

**Methods and analysis:**

Using a retrospective cross-sectional design, ViCS will examine trauma by violent crimes from 2000 to 2019. Data will be sourced from several institutions, including hospital records from nine emergency hospitals, and the National Board of Forensic Medicine Agency. The project aims to study medical and criminological aspects of violent crimes, primarily focusing on assaults involving firearms, sharp weapons, blunt instruments, kicks, punches, and other types of assault like strangulation. Data analysis will involve descriptive and inferential statistics.

**Discussion:**

ViCS aims to contribute to the limited body of knowledge about victims of violent crimes in Sweden. The findings may inform evidence-based interventions in medical, forensic, and criminological fields, potentially enabling targeted prevention strategies and improvements in emergency care for victims.

## 1. Background

In the recent decade, the homicide in Sweden has increased steadily. In 2012, Sweden witnessed its lowest rate of homicide (*n* = 68) since beginning of statistic records in 2002 by Swedish National Council for Crime Prevention. Since then, the rate of homicide has increased and reached a rate of 116 in 2022 (1). While the average annual rate of homicide in Sweden in 2020 was 1.20 per 100,000 inhabitants, the same figure for the European Union was 0.91 (2).

**Figure 1:**
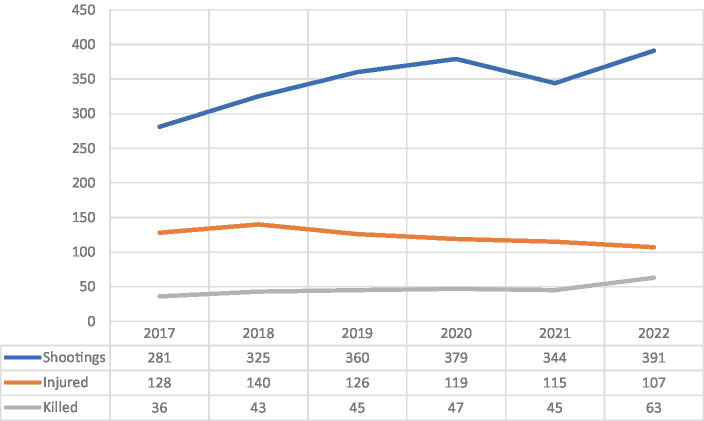
Trends in shooting incidents and associated injuries and fatalities in Sweden, 2017–2022.

Even though many types of violence, for example child homicide and homicide related to alcohol intoxication have decreased in Sweden (3, 4), one type of violence has increased. The firearm related violence, related to criminal gangs, is the single reason for the increase in homicide in Sweden (5, 6). One recent report from the Swedish National Council for Crime Prevention indicated that Sweden had on average 4 gun homicides per million inhabitants in comparison to an average of 1.6 in other European countries (7). During the last six years (2017–2022), Sweden has witnessed an annual average rate of 346.7 shootings, 122.3 individuals being injured, and 46.5 individuals being killed (8). Although the gun violence has been highly serious for a decade, the year of 2022 set a new record (Figure 1). With 391 confirmed shootings and 63 individuals being killed, Sweden witnessed its bloodiest year of gun-violence in modern time, as homicide by firearm caused 54% of all homicide in the country during that year (1).

While gun violence has spread all over Sweden, it has mostly affected the three largest cities, Stockholm, Gothenburg, and Malmö. For a long time, Malmö, located in region Skåne, had the highest rate of gun violence per resident and was called the Chicago of Sweden (9). While sharp force such as knives were the most common weapons to be used in Sweden during the 2010s, firearms was the weapon of choice in homicide in Skåne. Skåne, has also had the country’s second-highest rate of homicide (1.3 per 100,000 individuals) with only Stockholm (1.4 per 100,000 individuals) having more on average during the years 2014–2017 (10, 11). Gun violence is a new phenomenon in Sweden and has so far received little attention from the scientific community.

A systematic review by Khoshnood from 2018, revealed that only 25 articles had been published discussing rate, modus operandi, homicide typology, injuries and causes of death with respect to firearm-related violence in Sweden (12). Many of the included studies did not primarily focus on gun violence and included only specific types of cohorts or small cohorts. A minority (*n* = 9) of the included articles were focused on the medical aspects of gun violence and only three of them were published after 2015, the year when the gun violence increased considerable in Sweden.

Although gun violence has increased, at least up until 2017, knife was the most common weapon used in homicides for both men and women and remains a common modus operandi with respect to violent crimes (13). The rate of individuals being hospitalised for knife injuries have increased during the last decade and was in 2021 four times more common than hospitalisation because of firearm injuries (≈ 380 individuals respective ≈ 80 individuals) (14).

Despite penetrating trauma being the most common cause of homicide, assault with blunt force such as kicks, punches and blows with blunt instruments as well as other blunt force assaults, for example strangulation, remains the most common reported violent crimes. The total number of reported assaults to the police, including non-lethal penetrating violence, have varied between about 80,000 to 89,000 cases between 2013 and 2022, with the lowest rate seen in 2013. For 2022, 84,011 assault cases were reported to the police. The statistics show an increasing trend in reported assaults towards female victims, with a 15% increase in 2022 compared to 2013 (15). The majority (80%) of assaults against women were committed by perpetrators who had pre-existing relationships with the victim, such as close relatives or acquaintances (16).

With high rates of penetrating violence as well as reported assault rates, and the Swedish scientific production being highly limited, there is a major and urgent need for scientific studies mapping violent crimes in Sweden in general and Skåne in particular. The Violent Crimes in Skåne (ViCS) project aims to study violent crimes requiring hospitalization or violent crimes causing death in Skåne for the last 20 years with respect to medical, forensic, and criminological aspects. In this present paper, we describe the design of this large and multi-disciplinary project.

## 2. Materials and methods

### 2.1. Study setting

The project is set in the region Skåne, which is the most southern out of 21 regions in Sweden. Skåne is in close proximity to Denmark, separated only by the inlet of Öresund and connected by the Öresund bridge (7.8 km), that crosses it. With a population of 1.4 million, it is the third largest region in Sweden, which has a total population of 10.4 million. The largest city in Skåne is Malmö with its 351,000 inhabitants, followed by Helsingborg with 150,000 inhabitants.

Region Skåne has during the last two decades witnessed a significant population growth, much due to migration. The population over the age of 70 has increased markedly and is believed to continue increasing. The region is one of the most densely populated regions of Sweden with the population concentrated to the southwest (17).

**Figure 2:**
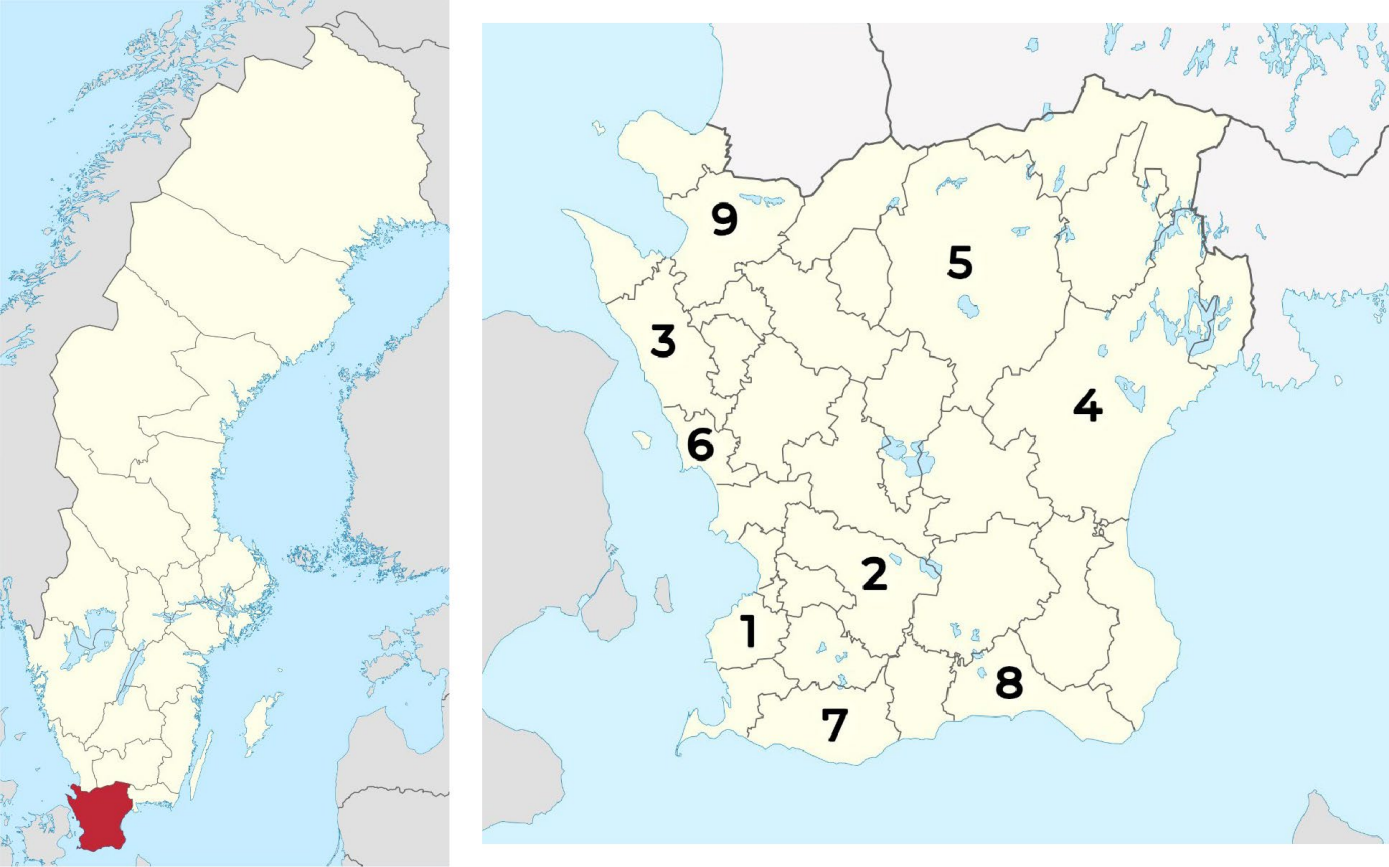
Region Skåne is prominently displayed on the left-side map. The right-side map provides the locations of the nine participating hospitals: (1) Skåne University Hospital Malmö; (2) Skåne University Hospital Lund; (3) Helsingborg Hospital; (4) Kristianstad Hospital; (5) Hässleholm Hospital; (6) Landskrona Hospital; (7) Trelleborg Hospital; (8) Ystad Hospital; and (9) Ängelholm Hospital. Swedish map with Region Skåne highlighted is by Wikimedia Commons, CC BY-SA 3.0, source: https://en.wikipedia.org/wiki/File:Skåne_län_in_Sweden.svg. The Region Skåne map is by Erik Frohne, CC BY 3.0, modified (numbers added to the map) from the original, source: https://en.wikipedia.org/wiki/File:Sweden_Scania_location_map.svg

Skåne has nine emergency hospitals: the university hospitals in Lund and Malmö, and the county hospitals of Helsingborg, Ystad, Kristianstad, Hässleholm, Trelleborg, Landskrona, and Ängelholm (Figure 2). Neurosurgery and thoracic surgery are focused to Lund while vascular and plastic surgery are focused to Malmö.

The ambulance organisation in Skåne is divided into four districts, all containing several ambulance stations. Two of the districts are run by the Region Skåne and two are run by a contractor procured by Region Skåne. All ambulances are staffed around the clock with at least two trained medical professionals out of which at least one is a specialist trained nurse (18).

### 2.2. Study design and data collection

The project is retrospective cross sectional, studying trauma caused by violent crimes within a 20-year time frame, from 2000 to 2019. The project is divided into sub-projects where we will study penetrating violence (firearm related violence and violence with sharp weapons) as well as blunt violence (assault with kicks and punches, assault with blunt instruments and other types of assault, like for example strangulation).

After receiving consent from Samråd KVB, which is a department in the Region Skåne deciding on disclosure of personal data for the purpose of research, personal identity numbers of patients treated at one of the nine mentioned hospitals during the study period and given one of the predetermined ICD-codes (Supplementary material), were acquired from the Department of Data and Analysis, which is responsible for providing patient data for research. The personal identity numbers were used to access medical records from the electronic medical record system, Melior, and from medical record archives.

Data regarding deceased victims, both treated at hospital before death and deceased prehospitally, were acquired from the Swedish National Board of Forensic Medicine (NBFM). The NBFM is a national governmental agency responsible for ordinances in forensic medicine, including medicolegal autopsies and clinical forensic medicine such as documentation and assessments of injuries on victims and suspected perpetrators. The NBFM performs all medicolegal non-natural death autopsies in Sweden, but only part of the ordinances in clinical forensic medicine. The board is subordinated the Department of Justice.

The Department of Forensic Medicine has six divisions that are dispersed throughout Sweden. The Division of Forensic Medicine in Lund is responsible for conducting forensic autopsies on suspected non-natural deaths, including homicides, within the borders of Skåne.

Data with respect to ethnicity were retrieved from the Swedish Tax Agency in the form of country of birth for the victim as well as his or her parents.

To ensure comprehensive data collection and avoid the omission of any individuals in the context of firearm-related violence, we also sourced data from the Swedish Police Region South including personal identity numbers of individuals victimized by firearm or sharp weapons. This region includes Skåne and allows us to cross-verify information collected from hospital records, thereby ensuring a thorough and accurate analysis.

The data variables collected are listed in Tables 1, 2.

**Table 1:** Variables studied in respect to modus operandi.

Variables	Definitions
**Firearm victimization**	
Entry wound	As described by the doctor’s examination or during surgery and noted in the medical records
Exit wound	As described by the doctor’s examination or during surgery and noted in the medical records
Shooting distance	The presumed distance between the offender and the victim at the time the shot was fired, if noted in the medical records
Type of weapon	The presumed firearm used in the crime, such as Automatic, Semi-Automatic, etc., if noted in the medical records
**Victimization by stabbing**	
Entry wound	As described by the doctor’s examination or during surgery and noted in the medical records
Type of weapon	The presumed sharp object used in the crime, such as knife, sword, etc., if noted in the medical records
**Victimization by assault not committed by a firearm or a sharp object**	
Type of assault	Includes assault with kicks and punches, assault with objects, as well as unspecified assault
Type of instrument	The specific object or tool used during the act of violence

**Table 2:** Variables studied in relation to all types of victimization.

Variables	Definitions/measurements
Sex	Male or female
Age	Measured in years
Procedure coding system	Based on ICD-10
Date of arrival	YYYYMMDD
Day of week of arrival	Monday, tuesday, *etcetera*
Time of arrival	First recorded time of arrival to ED, HH:MM
Date of discharge	YYYYMMDD
Place of discharge	Home, other hospital, nursing home, rehabilitation, or deceased
Diagnoses related to injury or in-hospital care	Based on ICD-10
Date of death	YYYYMMDD
Cause of death	The specific condition leading to the patient’s death
Place of injury	City or community (Malmö, Lund, and Helsingborg are considered cities, elsewhere is considered community)^a^
Area of injury	Private or common area (Private refers to private property, Common area refers to all other places, including in vehicles)
Distance from ambulance pick-up to hospital	Measured in km from site of ambulance pick-up to hospital, shortest route according to google maps
Hospital(s)	Any of the nine included hospitals that cared for the patient
Type of transport	Ambulance, private, or other (Other refers to other authority, e.g., police, or common transport, e.g., bus)
Time of transport departure from site	HH:MM
Ethnicity	Country of birth of patient and parents, according to the Swedish tax agency
Revised trauma score (RTS)	Calculated from GCS, systolic blood pressure, and respiration rate
Respiration rate	First measure recorded from arrival but within one hour, measured in breaths/min
Oxygen saturation	First measure recorded from arrival but within one hour, measured in Percent
Pulse	First measure recorded from arrival but within one hour, measured in heartbeats per minute
Blood pressure	First measure recorded from arrival but within one hour, systolic/diastolic measure in mmHg
Reaction level scale (RLS)	First measure recorded from arrival but within one hour, graded 1–8
Glasgow coma scale (GCS)	First measure recorded from arrival but within one hour, graded 3–15^b^
Body temperature	First measure recorded from arrival but within one hour, measured in Celsius
Haemoglobin	First measure recorded from arrival but within one hour, measured in mmol/L
Lactate	First measure recorded from arrival but within one hour, measured in mmol/L
Previous or ongoing contact with psychiatric clinic	Yes, no, or unknown
Psychiatric diagnosis	According to ICD-10
Drug influence	Measured by blood sample or noted in medical records from the current medical occasion
Injuries	According to medical records (ICD-10) or ICD-9 for Forensic Investigation
Specialties involved	Medical specialties involved due to injury or related diseases, other than emergency medicine physician
Level of care	Highest level recorded as ED, emergency ward, ward, or ICU^c^
Days of in-hospital care	Counted from arrival
Days of care in ICU	Counted from admittance to the ICU
Treatment in ED	As noted in medical records: blood transfusion, antibiotics, intubation, CPR, plaster, drainage
Treatment in hospital	As “Treatment in ED” plus surgery and wound care
ICD-code of surgery	Type of surgery conducted according to registered ICD-10 codes
CPR at arrival	Yes or No
Death in ED	Yes or No
Death in hospital	Yes or No

CPR, cardio pulmonary resuscitation; ED, emergency department; ICU, intensive care unit.

a This distinction aims to highlight the disparity between criminal activities within and outside of major cities. The incidence of violent crimes is progressively extending from urban areas to more isolated locations.

b In Sweden, the RLS-85 scale has traditionally been the exclusive measure for assessing levels of consciousness and continues to be widely used. When only the GCS-15 scale has been utilized, we will convert the results to the RLS-85 scale. However, when only the RLS-85 scale has been employed, we will not be able to convert the results to the GCS-15 scale due to its greater precision.

c Within a Swedish hospital, the distinction between an emergency ward and a general ward lies in the capacity for patient monitoring. The emergency ward can conduct more intensive monitoring through continuous blood pressure, pulse, and oxygen saturation measurements, as well as telemetry and repeated blood gas analyses. The emergency ward also maintains a higher staff-to-patient ratio compared to a general ward and can provide more advanced treatments.

### 2.3. Study objectives

The primary objective of this project is to study the injuries and causes of death among victims of violent crimes in Skåne between 2000 and 2019. Specifically, five types of violent crimes will be studied: (1) assault with firearms, (2) assault with sharp weapons/knives, (3) assault with blunt instruments, (4) assault with kicks and punches, and (5) other types of assault, like strangulation.

The research questions that will guide this project can be broadly grouped into medical, forensic, and criminological objectives with focus on, among others, epidemiology including the trend of violent crimes between 2000–2019, injury profile, cause or causes of death, treatments and patient outcome, as well as victim characteristics including sociodemographics.

### 2.4. Data analysis

While descriptive statistics like frequencies and medians will be used to discuss the characteristics of the study cohort, inferential statistical test like the chi-square test will be used to show the relationship between different categorical variables. Time trend analyses will be used to observe changes over time in our 20-year study period and understand the evolution of violent crime rates. To assess relationships between independent and dependent variables, for example types of violent crime and patient outcome, regression analyses will be used. Statistical significance, *p*-values and confidence intervals will be used to assess the reliability of the connections and results.

### 2.5. Ethics and dissemination

The ViCS project, including the waiver of informed consent, has been approved by the Swedish Ethical Review Authority (D.nr. 2020-02940). Before the start of the project, information about the study to the public was published at the website of Lund University.^1^ Victims are thereby given an opt-out opportunity and are informed that they can at any time and without further explanation discontinue their participation in the project.

The personal identity numbers and data related to the study objects are kept in a protected server and in a safe only accessible by the research team. All data will be registered into an Excel file, while at the same time being pseudonymized by giving each patient a unique ID number. All data processing and analysis will be conducted on the pseudonymized files. By analysing thousands of patients and presenting the results on a group level, we minimize the risk of individual victims being identified in published results.

Data collection is planned to be completed in the first six months of 2024. Data processing and analysis are expected to conclude in the second half of 2024, after which papers will begin to be published. The results will also be highlighted at various medical congresses.

Among the variables examined in our research, ethnicity—or nationality—merits an in-depth exploration due to its profound implications in both criminology and medicine. Within the criminological sphere, ethnicity emerges as a complex indicator linked to routine activities, living conditions, and socioeconomic status, all of which collectively contribute to the likelihood of becoming a victim of violent crime (19–24). Parallel to its role in criminology, ethnicity holds significant value in the medical field. Existing literature, highlights alarming ethnic disparities, not least in the realm of trauma care (25). For instance, Chang et al. showed that black patients experience a heightened risk—up to 20%—of adverse events relative to non-black patients (26).

## 3. Discussion

The immense increase in gun violence in Sweden is highly troublesome. Efforts have been made, albeit without success, to suppress the criminal gangs and criminal networks in the country (12, 27, 28). It is unclear why Sweden has such serious problems with firearm related violence in comparison to both neighbouring countries as well as the rest of the western world. Factors like increased gang criminality, high availability to illegal firearms, the existence of open drug markets as well as vulnerable areas, have been mentioned (5–7, 12, 29, 30). The gun violence in the country has thus created some serious challenges for the law enforcement, the judicial system, Swedish politicians and not least, the Swedish emergency care (5, 31).

Despite the increase in penetrating violence, homicide, and the high rates of reports regarding other violent crimes in Sweden, not least with respect to females, the scientific literature is sparse. In discussing individuals being involved in gun violence, the absolute majority of the published studies are focused on the offenders [e.g., (29, 32–34)]. There are few studies published during the last years which in depth try to discuss penetrating trauma with respect to the victims (35–37). All have contributed to our knowledge, but have some limitations, such as a small number of victims studied, few data sources, or a limited time frame.

By looking into the epidemiology and medical aspects of the victims of violent crimes during a 20 years’ time frame, during which the homicide increased steeply, we aim to increase knowledge about these crimes, the injuries and cause of death and the victims themselves.

The results from the ViCS-project regarding, among other things, the epidemiology, victims and medical aspects of violent crimes could contribute to evidence-based interventions on both the medical and criminological fronts. Contributions to both fields expand our understanding on multiple levels.

With regard to gun violence, the knowledge of the victims, i.e., victimology, can be used to find common characteristics contributing to targeted prevention strategies.

The project may help develop specialized procedures, training programs, or resource allocation techniques that enhance the quality of care for these patients by analysing injuries, treatments, patient outcomes and causes of death (visual abstract).

### 3.1. Strengths and limitations

The project will be conducted in the third largest region in Sweden, providing a large underlying population and number of victims. Studying a long timeframe will make it possible to identify trends. Using multiple data sources (the hospital, the police, the NBFM etc.) is another strength of this project. During this long timeframe, however, criminal activity has increased and decreased in cycles, as a consequence of criminal activity moving between cities and regions and crime preventing interventions being introduced and discontinued. Also, during this time frame, the hospital organisation has changed markedly, making comparison between time periods more difficult. Being a retrospective medical record study, another limitation of this project is the dependence on the quality of the data recorded.

## Scope statement

Our manuscript, titled “Understanding Violent Crimes in Sweden’s Region Skåne: a Retrospective Cross-Sectional Design of the ViCS Project”, aligns closely with the mission and scope of Frontiers in Psychiatry. The study dives deep into the epidemiology of violent crimes, a pressing public health issue, and aims to uncover underlying causes and potential preventive strategies. This focus directly resonates with the journal’s commitment to investigating the causes of health states and diseases and its emphasis on methodological rigor and data practices. By providing a comprehensive analysis that spans nearly two decades, we aim to contribute to the journal’s objective of improving health at both clinical and population levels.

## Ethics statement

The project involving human participants was approved by the Swedish Ethical Review Authority. It was conducted in accordance with local legislation and institutional requirements. Written informed consent for participation was not required from the participants or their legal guardians/next of kin, as per approval by the Swedish Ethical Review Authority.

## Author contributions

AR: Data curation, Investigation, Project administration, Writing – original draft. BA: Data curation, Investigation, Project administration, Writing – original draft. CW: Data curation, Methodology, Writing – review & editing. UE: Data curation, Methodology, Writing – review & editing. AK: Data curation, Methodology, Conceptualization, Funding acquisition, Investigation, Project administration, Resources, Supervision, Writing – original draft.

## Footnotes

^1^ https://www.lupop.lu.se/valdsbrott-i-skane
