# Supplementary material for: Understanding medical aspects of violent crimes in Sweden’s region Skåne: a retrospective cross-sectional design of the ViCS project
Source: Front Psychiatry. 2023 Nov 9;14:1287007. doi: 10.3389/fpsyt.2023.1287007 (PMC10665833; doi:10.3389/fpsyt.2023.1287007)
Supplement: Supplementary file 2 [file Data_Sheet_1.docx]

**Additional File – ICD-codes**

**Gun violence**

- W32 Handgun discharge
- W33 Rifle, shotgun and larger firearm discharge
- W34 Discharge from other and unspecified firearms
- X93 Assault by handgun discharge
- X94 Assault by rifle, shotgun and larger firearm discharge
- X95 Assault by other and unspecified firearm discharge
- Y22 Handgun discharge, undetermined intent
- Y23 Rifle, shotgun and larger firearm discharge, undetermined intent
- Y24 Other and unspecified firearm discharge, undetermined intent
- Y35.0 Legal intervention involving firearm discharge

**Sharp weapons**

- W25 Contact with sharp glass
- W26 Contact with other sharp object(s)
- X99 Assault by sharp object
- Y28 Skadehändelse med skärande eller stickande föremål, med oklar avsikt
- Y35.4 Contact with sharp object, undetermined intent

**Assault**

- X85 Assault by drugs, medicaments and biological substancesX86 Övergrepp med frätande ämnen
- X87 Assault by pesticides
- X88 Assault by gases and vapours
- X89 Assault by other specified chemicals and noxious substances
- X90 Assault by unspecified chemical or noxious substance
- X91 Assault by hanging, strangulation and suffocation
- X92 Assault by drowning and submersion
- X96 Assault by explosive material
- X97 Assault by smoke, fire and flames
- X98 Assault by steam, hot vapours and hot objects
- Y00 Assault by blunt object
- Y01 Assault by pushing from high place
- Y02 Assault by pushing or placing victim before moving object
- Y03 Assault by crashing of motor vehicle
- Y04 Assault by bodily force
- Y07 Other maltreatment
- Y08 Assault by other specified means
- Y09 Assault by unspecified means
- Y25 Contact with explosive material, undetermined intent
- Y29 Contact with blunt object, undetermined intent
